# LINC00052 upregulates EPB41L3 to inhibit migration and invasion of hepatocellular carcinoma by binding miR-452-5p

**DOI:** 10.18632/oncotarget.18892

**Published:** 2017-06-29

**Authors:** Liying Zhu, Nenghong Yang, Juan Chen, Tao Zeng, Shaoying Yan, Yuyang Liu, Gangfeng Yu, Qiuxu Chen, Guiqin Du, Wei Pan, Xing Li, Huihao Zhou, Ailong Huang, Hua Tang

**Affiliations:** ^1^ Key Laboratory of Molecular Biology for Infectious Diseases (Ministry of Education), Institute for Viral Hepatitis, Department of Infectious Diseases, The Second Affiliated Hospital, Chongqing Medical University, Chongqing, China; ^2^ Collaborative Innovation Center for Diagnosis and Treatment of Infectious Diseases, Zhejiang University, Hangzhou, China; ^3^ Department of Medical Laboratory, Guizhou Medical University, Guiyang, China; ^4^ Department of Hepatobiliary Surgery, Affiliated Hospital of Guizhou Medical University, Guiyang, China

**Keywords:** HCC, LINC00052, EPB41L3, migration, invasion

## Abstract

Numerous studies have demonstrated that a class of long noncoding RNAs (lncRNAs) are dysregulated in hepatocellular carcinoma (HCC) and they are closely related with tumorigenesis. Our previous studies indicated that LINC00052 was a downregulated lncRNA in HCC and acted as a tumor suppressor gene. Using transcription microarray analysis, we found that knockdown of LINC00052 resulted in EPB41L3 downregulation. However, the function of EPB41L3 and the mechanism of LINC00052 downregulating EPB41L3 in HCC remain unclear. In this study, we found that overexpression of LINC00052 could upregulate the EPB41L3 expression and it might serve as a tumor suppressor gene in HCC. Database analysis showed that miR-452-5P could target LINC00052. The binding regions between LINC00052 and miR-452-5P were confirmed by luciferase assays. Moreover, LINC00052 inhibited cell malignant behavior by increasing miR-452-5P expression, suggesting that LINC00052 was negatively regulated by miR-452-5P. In addition, overexpression of miR-452-5P resulted in a decrease of EPB41L3 expression, suggesting that EPB41L3 was as a target of miR-452-5P. In conclusion, these results demonstrated that a novel pathway was mediated by LINC00052 in HCC.

## INTRODUCTION

Hepatocellular carcinoma (HCC) is one of the most common liver cancers worldwide, particularly in Asian areas. Despite of the therapeutic advances in HCC in the past few decades, HCC has resulted in a very high death rate [1, 2]. The process of HCC is involved in complicated steps. Intensive data from whole genome and transcriptome studies have revealed that only 1-2% of the genome encodes proteins, but the majority of the mammalian genome encodes large numbers of noncoding RNAs [3, 4]. Among them, long non-coding RNAs (lncRNAs) are defined as transcribed RNA molecules and they are more than 200nt in length with limited or no protein-coding capacity [5]. Recent studies have demonstrated that various lncRNAs, such as UCA1, ATB, Dreh, HOTAIR and HOTTIP, have been implicated in cell proliferation, apoptosis and metastasis of liver cancer cells [6–10].

Our previous studies indicated that LINC00052 was a downregulated lncRNA in HCC [11]. We found that erythrocyte membrane protein band 4.1-like 3(EPB41L3)(NCBI Accession NO. NM_001281534.1) was lowly expressed in A554 cells compared with control cells (SMMC7721) by transcriptome microarray analysis (Supplementary Table 1). EPB41L3, which belongs to the protein 4.1 family [12], is a membrane skeletal protein. EPB41L3 is extensively expressed in various human tissues. However, downregulation of EPB41L3 was frequently observed in cases of gastric cancer, non-small cell lung carcinoma cells and renal clear cell carcinoma [13–15]. This evidence suggests that EPB41L3 acts as a tumor suppressor in pathogenesis of these tumors. Liang and his colleagues also reported that EPB41L3 overexpression decreased cell invasion in the A549 lung cancer cell while EPB41L3 knockdown increased cell invasion [16]. Accumulating studies have demonstrated that EPB41L3 overexpression inhibited the migration and invasion of NSCLC cells [14, 17]. However, the underlying mechanism of LINC00052 upregulating EPB41L3 in HCC remains unclear.

MicroRNAs (miRNAs) were a class of conserved, small non-coding RNAs, approximately 22 nucleotides, which could inhibit gene expression by binding in 3′ untranslated region (3′ UTR) of the target mRNAs [18–20]. It was reported that miR-452 was up-regulated in HCC, renal cell carcinoma, esophageal cancer, urothelial carcinoma and prostate cancer stem cells [21–25]. It was confirmed that LINC00052 harbored two miR-452-5P binding sites by bioinformatics databases (BiBi Serv). Moreover, using miRNA target prediction software, EPB41L3 was predicted to be a presumed target of miR-452-5P. According to the recently proposed competing endogenous RNA hypothesis, multiple lncRNA transcripts can act as endogenous decoys for miRNAs through their miRNA binding sites, and thus may reciprocally repress the biological activity of one another.

In the present study, we investigated the expressions of LINC00052, EPB41L3 and miR-452-5P, and analyzed their functions in human HCC tissues and HCC cell lines. We found that miR-452-5P negatively regulated LINC00052 by directly targeting the miRNA-binding sites in LINC00052. These results illustrated that LINC00052 might act as a molecular sponge for miR-452-5P and downregulated its downstream gene of EPB41L3 in HCC.

## RESULTS

### LINC00052 was downregulated in HCC and overexpression of LINC00052 inhibited HCC cell proliferation, migration and invasion *in vitro*

**Figure 1:**
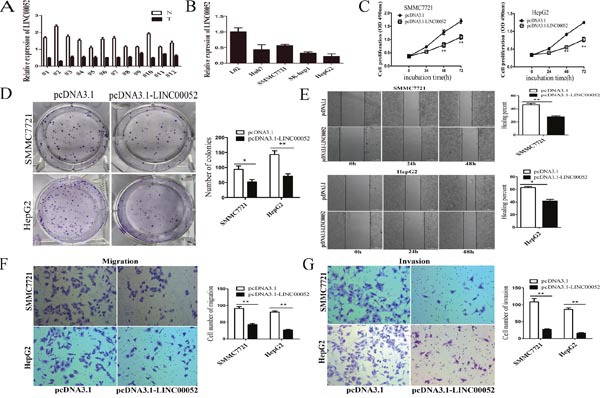
LINC00052 was downregulated in HCC and overexpression of LINC00052 inhibited HCC cell proliferation, migration and invasion *in vitro* **(A)** LINC00052 expression in 12 pairs of HCC tissues (T) and non-tumor tissues (N). Transcript levels were normalized to GAPDH expression. **(B)** LINC00052 expression in L02 cells and four cell lines. Transcript levels were normalized to GAPDH expression. **(C)** Growth curves of SMMC7721 and HepG2 cells after transfection with pcDNA3.1-LINC00052 or pcDNA3.1. **P<0.01. **(D)** Colony formation assays determining the effect of LINC00052 upregulation on the growth of SMMC7721 and HepG2 cells. Representative graphs are shown. The data graphs depict the count number from three independent experiments. *P<0.05, **P<0.01. **(E)** Wound healing assay for determining the effect of LINC00052 upregulation on the healing of SMMC7721 and HepG2 cells. *P<0.05, **P<0.01. **(F, G)** Transwell and invasion assay of LINC00052 overexpressed cells. Data are shown as mean ± s.d. (n = 3) and are representative of three independent experiments. Scale bars = 100 μm. **P<0.01 (Student's t-test).

We firstly examined the expressions of LINC00052 in human HCC tissues, and found that LINC00052 was lower expressed in 12 paired HCC tissues than non-tumour tissues (Figure 1A). Then the expressions of LINC00052 in several HCC cell lines were detected. LINC00052 was also significantly decreased in HCC cell lines (SMMC7721, Huh7, SK-hep1and HepG2) comparison with that in immortalized, normal human liver cell line (L02) (Figure 1B). We selected SMMC7721 and HepG2 cells for functional experiments. Overexpression of LINC00052 inhibited cell growth by using MTS assays (Figure 1C). In colony formation assays, the colony number was much lower in the LINC00052 ectopically overexpressing cells compared with control cells (Figure 1D). In wound healing assays, LINC00052 overexpression decreased migratory capacity (Figure 1E). Transwell assays confirmed that ectopic overexpression of LINC00052 significantly reduced the number of cells crossing the membrane (Figure 1F). Moreover, we investigated invasive ability alterations by using matrigel-coated transwell experiments and found fewer cells invading through when overexpressing LINC00052 (Figure 1G). Taken together, these results indicated that overexpression of LINC00052 repressed the tumorigenicity of HCC cells.

### Inhibition of LINC00052 promoted HCC cell proliferation, migration and invasion

In order to precisely examine the roles of LINC00052 in HCC, HCC cell lines were transfected with siRNAs to inhibit the expression of LINC00052. MTS assays showed that LINC00052 knockdown promoted cell proliferation in both SMMC7721 and HepG2 cells (Supplementary Figure 1A). Colony formation assay confirmed that the numbers of colony were markedly increased following LINC00052 knockdown (Supplementary Figure 1B). In wound healing assays, migratory speed was enhanced after LINC00052 knockdown (Supplementary Figure 1C). Transwell assays showed that the number of cells crossing the membrane were increased (Supplementary Figure 1D). In addition, knockdown of LINC00052 promoted cell invasion in SMMC7721and HepG2 cells (Supplementary Figure 1E). Taken together, these results indicated that inhibition of LINC00052 increased the tumorigenicity of HCC cells.

### LINC00052 upregulated the expression of EPB41L3

In our previous studies [11], when SMMC77221 cells were transfected with gene trapping vector PU21 and selected by G418, lots of cell colonies were set up and then cultured into cell lines. Among them, a cell line (A554) had a stronger migration, invasion and proliferation ability comparison with SMMC7721 cells. RACE result showed that the gene trapped by PU21 in A554 cell line was LINC00052. And LINC00052 was knocked down in A554 cell line.

In order to investigate downstream target of LINC00052 in HCC, we carried out a microarray analysis (Supplementary Table 1) and found that knockdown of LINC00052(A554) resulted in downregulated expression of EPB41L3.

**Figure 2:**
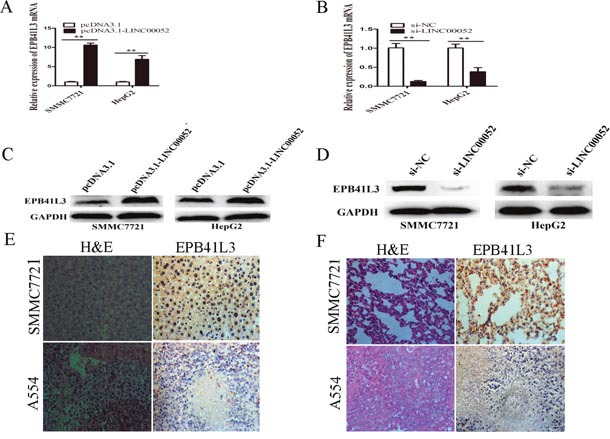
LINC00052 upregulated the expression of EPB41L3 **(A)** RT-qPCR analysis of EPB41L3 mRNA expression when SMMC7721 and HepG2 cells were transfected with pcDNA3.1-LINC00052 or pcDNA3.1 vector. Transcript levels were normalized to GAPDH expression. **P<0.01. **(B)** RT-qPCR analysis of EPB41L3 mRNA expression when SMMC7721 and HepG2 cells were transfected with si-LINC00052 or si-NC. Transcript levels were normalized to GAPDH expression. **P<0.01. **(C)** Western blot analysis of EPB41L3 protein expression when SMMC7721 and HepG2 cells were transfected with pcDNA3.1-LINC00052 or pcDNA3.1 vector, using GAPDH as an endogenous control. **(D)** Western blot analysis of EPB41L3 protein expression when SMMC7721 and HepG2 cells were transfected with si-LINC00052 or si-NC, using GAPDH as an endogenous control. **(E)** Hematoxylin and eosin (H&E) staining and immunohistochemical staining of EPB41L3 in the mouse liver of LINC00052 knockdown group compared with the control group *in vivo*. Representative micrographs were shown, original magnification (×400). **(F)** Hematoxylin and eosin (H&E) staining and immunohistochemical staining of EPB41L3 in the mouse lung of LINC00052 knockdown group compared with the control group *in vivo*. Representative micrographs were shown, original magnification (×400).

RT-qPCR and western blot assays were performed to confirm the effects of LINC00052 on the expression of mRNA and protein levels of EPB41L3. The mRNA expression levels of EPB41L3 in the cells transfected with pcDNA3.1-LINC00052 were higher than those cells transfected with pcDNA3.1 (Figure 2A). In contrast, the mRNA expression of EPB41L3 in the si-LINC00052 group was down-regulated compared with the si-NC group (Figure 2B). Similarly, EPB41L3 protein was markedly decreased in pcDNA3.1- LINC00052 transfected cells than that in control cells (Figure 2C), while EPB41L3 protein increased in si-LINC00052 transfected cells (Figure 2D). Immunohistochemistry assay showed weak or no expression of EPB41L3 in the liver and lung of mice in the LINC00052 knockdown group compared with the control group *in vivo* (Figure 2E, 2F). These results revealed that LINC00052 upregulated the expression of EPB41L3 *in vitro* and *in vivo*.

### EPB41L3 was significantly downregulated in HCC tissues and HCC cell lines

**Figure 3:**
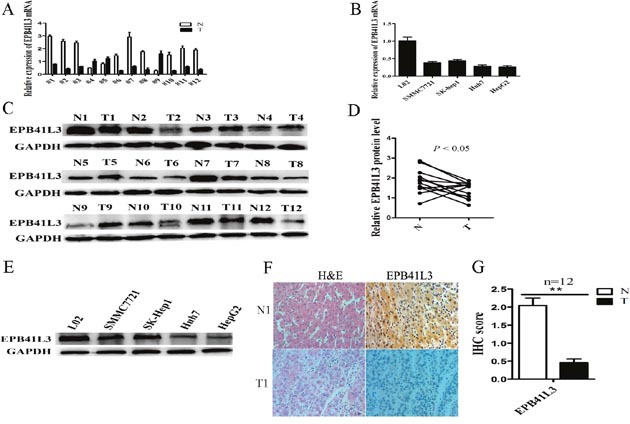
EPB41L3 was significantly downregulated in HCC tissues and HCC cell lines **(A)** RT-qPCR analysis of EPB41L3 expression in 12 pairs of HCC tissues (T) and non-tumor tissues (N). Transcript levels were normalized to GAPDH expression. **(B)** RT-qPCR analysis of EPB41L3 expression in L02 cells and four cell lines. Transcript levels were normalized to GAPDH expression. **(C)** Western blot analysis of EPB41L3 protein expression in 12 pairs of HCC tissues (T) and non-tumor tissues (N), using GAPDH as an endogenous control. **(D)** Quantitation of EPB41L3 protein expression levels by their integrated light density values. n = 12, *P<0.05. **(E)** Western blot analysis of LINC00052 expressions in L02 cells and four cell lines, using GAPDH as an endogenous control. **(F)** Hematoxylin and eosin (H&E) staining and immunohistochemical staining of EPB41L3 in HCC tissues (T) and non-tumor tissues (N). **(G)** Total Immunohistochemical (IHC) score of EPB41L3 in HCC tissues and non-tumour tissues (n = 12). **P<0.01.

Firstly, the expressions of EPB41L3 in HCC tissues and HCC cell lines were measured by RT-qPCR and western blot. The results showed that EPB41L3 were significantly lower in tumor tissues and HCC cell lines (Figure 3A–3E). Furthermore, immunohistochemistry assay revealed that the EPB41L3 protein was differentially expressed between human tumour tissues and their matched non-tumour tissues. Strong immunoreactivity for EPB41L3 was detected along the cell membrane and in the cytoplasm of the non-tumour tissues. However, the HCC tissues showed weak or no expression of EPB41L3 (Figure 3F, 3G).

### EPB41L3 repressed HCC cell migration and invasion *in vitro*

**Figure 4:**
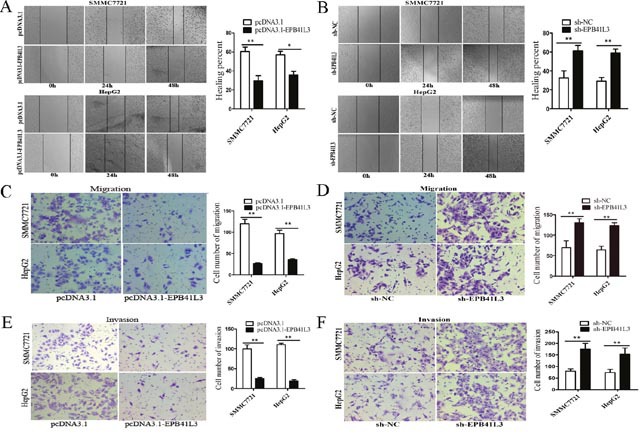
EPB41L3 repressed HCC cell migration and invasion *in vitro* **(A, B)** Wound healing assay were performed to determine the healing ability of EPB41L3 in SMMC7721 and HepG2 cells. *P<0.05, **P<0.01. **(C-F)** The effect of EPB41L3 on the migration and invasion of SMMC7721 and HepG2 cells was assessed using transwell assays. **P<0.01.

Next, we explored the function of EPB41L3 in HCC. Tumor migration and invasion are important steps in tumor progression. Therefore, we first investigated the role of EPB41L3 in HCC cell migration and invasion. In wound healing assays, EPB41L3 overexpression decreased migratory capacity (Figure 4A). In contrast, migratory speeds were enhanced after EPB41L3 were knockdown (Figure 4B). Transwell assays found the EPB41L3 decreased HCC cell migratory capacity (Figure 4C), while EPB41L3 knockdown increased cell migration (Figure 4D). In matrigel-coated transwell chamber assays, fewer cells invaded through when overexpressing EPB41L3 (Figure 4E). And knockdown of EPB41L3 showed opposite effects (Figure 4F). Taken together, EPB41L3 inhibited the migration and invasion of HCC cells *in vitro*.

### EPB41L3 repressed HCC metastasis *in vivo*

**Figure 5:**
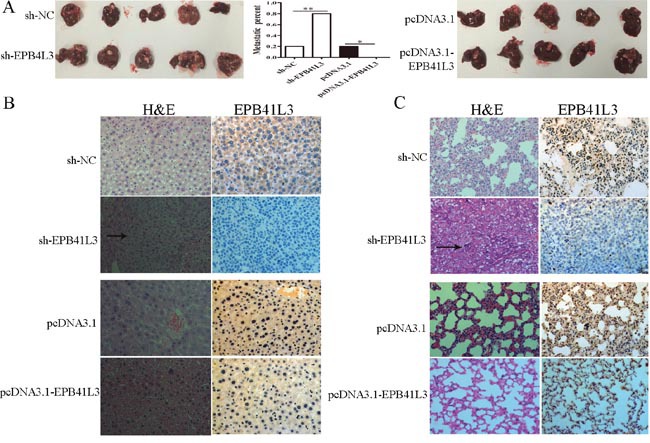
EPB41L3 repressed HCC metastasis *in vivo* **(A)** Livers of metastases 6 weeks after orthotopic implantation stably overexpressed or interference EPB41L3 SMMC7721 cells (n=5). *P<0.05, **P<0.01. **(B)** H&E staining and immunohistochemical staining of EPB41L3. Representative photograph of livers. Black arrow: metastatic foci. **(C)** H&E staining and immunohistochemical staining of EPB41L3. Representative photographs of lung. Black arrow: metastatic foci.

To further probe the effects of EPB41L3 *in vivo*, we constructed four SMMC7721 cell lines: pcDNA3.1-EPB41L3 stably expressing EPB41L3 cells, pcDNA3.1 stably expressing cells, sh-EPB41L3 stably expressing and sh-NC expressing cells. Then, these cells were injected into the liver of nude mice, respectively. Six weeks later, we found more metastatic nodules in the livers of mice in sh-EPB41L3 group(4 of 5 mice) compared with its control group (1 of 5 mice). Moreover, less metastatic nodules were found in livers of pcDNA3.1-EPB41L3 group(0 of 5 mice) compared with its control pcDNA3.1 group (1 of 5 mice) (Figure 5A). The histopathological analysis showed that in sh-EPB41L3 group, an increasing number of lymphocytes were found in liver and lung tissue compared with its control group (sh-NC). In pcDNA3.1-EPB41L3group, no lymphocytes were found in liver and lung tissue compared with its control pcDNA3.1 group, and these results were further confirmed by immunohistochemistry assay (Figure 5B, 5C). The above data revealed that EPB41L3 repressed HCC migration and invasion *in vivo*.

### Reciprocal repression between LINC00052 and miR-452-5P

**Figure 6:**
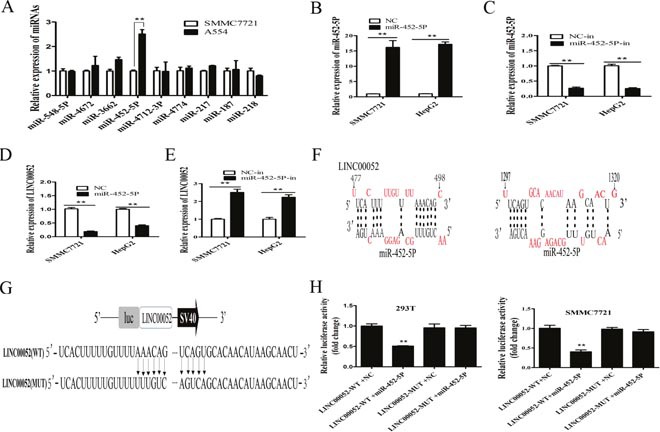
Reciprocal repression between LINC00052 and miR-452-5P **(A)** Several miRNAs expressions in HCC cells that stably knocked down LINC00052(A554) and SMMC7721, using U6 as an internal control. **P<0.01. **(B)** RT-qPCR analysis of miR-452-5P expression in SMMC7721and HepG2 cells transfected with miR-452-5P mimic (miR-452-5P) or NC, using U6 as an internal control. **P<0.01. **(C)** RT-qPCR analysis of miR-452-5P expression in SMMC7721and HepG2 cells transfected with miR-452-5P inhibitor (miR-452-5P-in) or inhibitor NC (NC-in), using U6 as an internal control. **P<0.01. **(D, E)** miR-452-5P regulated the expression of LINC00052 in HCC cells. Relative expression levels of LINC00052 were detected by RT-qPCR, using U6 as an internal control. **P<0.01. **(F)** Predicted binding sites for miR-452-5P in LINC00052 sequences. Numbers show the nucleotides relative to the transcriptional start site of LINC00052. **(G)** Schematic of wild-type and mutant PGL3-LINC00052 constructs. **(H)** Luciferase assays of 293T and SMMC7721 cells transfected with PGL3-LINC00052-WT or PGL3-LINC00052-MUT reporter and NC or miR-452-5P mimic. **P<0.01.

How LINC00052 downregulated EPB41L3 in HCC? LncRNA might function as a competing endogenous RNA (ceRNA) in modulating the expression and biological functions of miRNA [26, 27]. According to this rule, several miRNAs as the potential targets of LINC00052 were predicted by the bioinformatics databases (DIANA-LncBase, miRcode, BiBi Serv), such as miR-452-5p, miR-548-5p, miR-4672, miR-3662. These miRNAs expressions in SMMC7721 and A554 cells were detected. Compared with other potential miRNAs, the expression of miR-452-5P was the highest in A554 cells (Figure 6A). For further analysis the relation between miR-452-5P and LINC00052, miR-452-5P mimic and miR-452-5P inhibitor (miR-452-5P-in) were used, and their overexpression or inhibition of miR-452-5P were confirmed by RT-qPCR (Figure 6B, 6C). Then, HCC cells were transfected with miR-452-5P mimic or miR-452-5P inhibitor, and the expression of LINC00052 was examined. Results showed that the expression levels of LINC00052 were decreased in miR-452-5P mimic group compared with miR-452-5P-NC group (Figure 6D), whereas the expression of LINC00052 were increased in miR-452-5P inhibitor (miR-452-5P-in) group compared with control NC-in group (*P*<0.01) (Figure 6E). These data showed that there was reciprocal repression between LINC00052 and miR-452-5P.

There were two miR-452-5P binding sites in LINC00052 sequence by bioinformatics analysis (Figure 6F). To explore the underlying mechanism of the lncRNA/miRNA regulatory function, dual-luciferase reporter assay was performed. We constructed luciferase reporters comprising the 1062 nt of LINC00052, which contained either wild-type (WT) or mutated (MUT) miR-452-5P binding sites (Figure 6G). As a result, miR-452-5P mimic reduced the luciferase activity of the LINC00052 WT reporter but not in NC or the mutated reporter (Figure 6H).

### MiR-452-5p was upregulated in HCC tissues and HCC cell lines, and functioned as an oncogene

**Figure 7:**
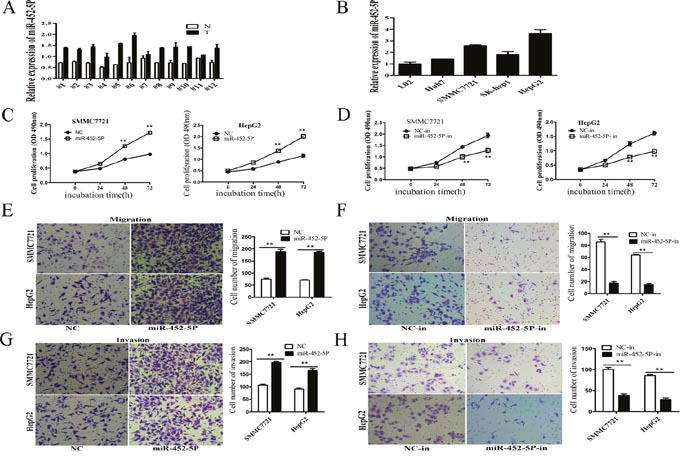
MiR-452-5p was upregulated in HCC tissues and HCC cell lines, and functioned as an oncogene **(A)** RT-qPCR analysis of miR-452-5P expression in 12 pairs of HCC tissues (T) and non-tumor tissues (N), using U6 as an internal control. **(B)** RT-qPCR analysis of LINC00052 expression in L02 cells and four cell lines, using U6 as an internal control. **(C)** Growth curves of SMMC7721 and HepG2 cells after transfection with miR-452-5P or NC were determined by MTS assays. **P<0.01. **(D)** Growth curves of SMMC7721 and HepG2 cells after transfection with miR-452-5P inhibitor (miR-452-5P-in) or inhibitor NC (NC-in), were determined by MTS assays. **P<0.01. **(E-H)** The effects of miR-452-5P on the migration and invasion of SMMC7721 and HepG2 cells were performed using transwell assays. **P<0.01.

RT-qPCR showed that miR-452-5P was upregulated in the 12 paired HCC tissues than non-tumour tissues (Figure 7A). Compared with L02 cells, HCC cell lines displayed higher expression of miR-452-5P (Figure 7B). Overexpression of miR-452-5P promoted cell growth in MTS assays (Figure 7C), whereas inhibited proliferation occurred after knockdown of miR-452-5P by miR-452-5P inhibitor (miR-452-5P-in) (Figure 7D). Transwell assays confirmed the promotion of miR-452-5P on HCC cell migratory capacity in both SMMC7721 and HepG2 cell lines (Figure 7E). However, knockdown of miR-452-5P significantly reduced the number of cells crossing the membrane (Figure 7F). In matrigel-coated transwell assays, more cells invading through when overexpression miR-452-5P (Figure 7G). Knockdown of miR-452-5P showed opposite effects (Figure 7H).

### MiR-452-5P recovered LINC00052 function in HCC cells

**Figure 8:**
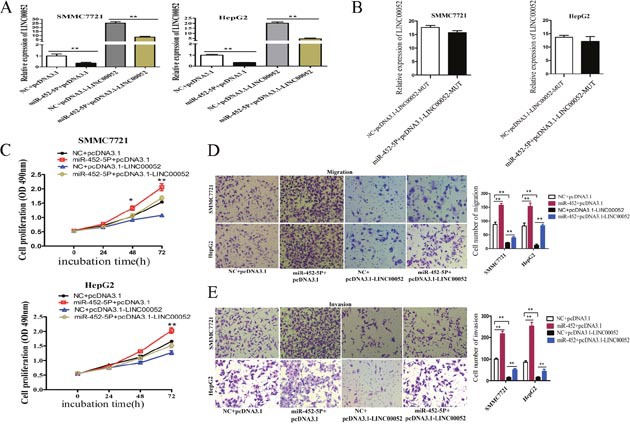
MiR-452-5P recovered LINC00052 function **(A)** SMMC77221 and HepG2 cells were co-transfected with miR-452-5P mimic and LINC00052 expression plasmid, and the effect of miR-452-5P on ectopically expressed LINC00052 was analyzed by RT-qPCR. **P<0.01. **(B)** SMMC77221 and HepG2 cells were co-transfected with miR-452-5P mimic and pcDNA3.1-LINC00052-MUT plasmid (with miR-452-5P mutant binding site), and the effect of miR-452-5P on ectopically mutant LINC00052 expression was analyzed by RT-qPCR. **P<0.01. **(C)** SMMC77221 and HepG2 cells were co-transfected with negative control or miR-452-5P mimic and control plasmid (pcDNA3.1) or LINC00052 expression plasmid (pcDNA3.1- LINC00052) and cell proliferations were determined using MTS assays. *P<0.05, **P<0.01. **(D, E)** Transwell migration and invasion assays of HCC cells after cotransfected with negative control or miR-452-5P mimic and control plasmid (pcDNA3.1) or LINC00052 expression plasmid (pcDNA3.1- LINC00052).**P<0.01.

SMMC7721 and HepG2 cells were co-transfected with miR-452-5P mimic and the vector expressing LINC00052 to study the effects of miR-452-5P on cell proliferation, migration and invasion mediated by LINC00052. As shown in Figure 8A, miR-452-5P could significantly decrease the LINC00052 expression in both cell lines, but nearly have no effect on the expression of mutant miR-452-5P (Figure 8B). MTS proliferation assays revealed that miR-452-5P promoted cell proliferation and LINC00052 suppressed cell proliferation, while co-transfection of miR-452-5P and LINC00052 expressing-plasmids, miR-452-5P could recover the effect of LINC00052 in suppressing cell proliferation (Figure 8C). Moreover, transwell migration and invasion assay demonstrated that miR-452-5P promoted and LINC00052 suppressed cell migration and invasion, while co-transfection of miR-452-5P mimic and LINC00052 expressing vector, miR-452-5P promoted cell migration and invasion inhibited by LINC00052 (Figure 8D, 8E). These observations suggested that the effects of LINC00052 overexpression on the promotion of SMMC7721 and HepG2 cell proliferation, migration and invasion could be recovered by miR-452-5P.

### MiR-452-5P inhibited the expression of EPB41L3 by targeting its 3′UTR

**Figure 9:**
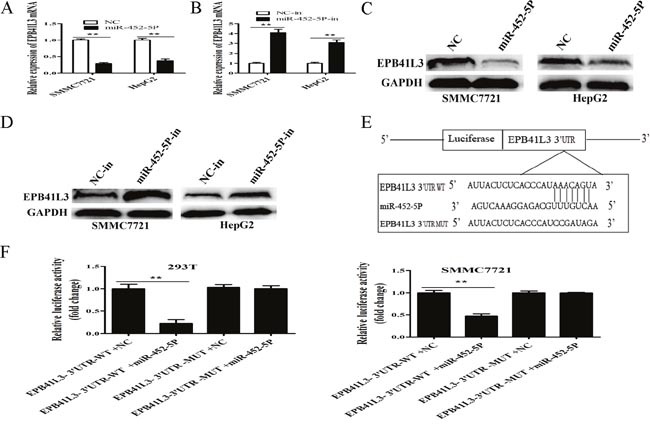
EPB41L3 was a direct target of MiR-452-5P **(A)** RT-qPCR analysis of EPB41L3 mRNA expression when SMMC7721 and HepG2 cells were transfected with miR-452-5P mimic or NC. Transcript levels were normalized to GAPDH expression. **P<0.01. **(B)** RT-qPCR analysis of EPB41L3 mRNA expression when SMMC7721 and HepG2 cells were transfected with miR-452-5P inhibitor (miR-452-5P-in) or inhibitor NC (NC-in). Transcript levels were normalized to GAPDH expression. **P<0.01. **(C, D)** miR-452-5P regulated the expression of EPB41L3 in HCC cells. Relative protein expression levels of EPB41L3 were detected by Western blot analysis, using GAPDH as an endogenous control. **(E)** Schematic of wild-type and mutant PGL3-EPB41L3-3′UTR constructs. **(F)** Luciferase assays of 293T and SMMC7721 cells transfected with PGL3-EPB41L3-3′UTR-WT or PGL3- EPB41L3-3′UTR-MUT reporter and NC or miR-452-5P mimic. **P<0.01.

Interestingly, EPB41L3 was the one of potential downstream target genes of miR-452-5P predicted by the bioinformatics databases (TargetScan, Pictar and Miranda). To experimentally verify the prediction, cells were transfected with miR-452-5P mimic or miR-452-5P inhibitor, the mRNA and protein expression levels of EPB41L3 levels were assessed by qRT-PCR and western blot assay. As a result, miR-452-5P inhibited expression of EPB41L3 both in mRNA and protein levels (Figure 9A–9D).

To further elucidate whether EPB41L3 was a functional target of miR-452-5P, dual-luciferase reporter assay was conducted in 293T and SMMC7721 cells. The binding sites of miR-452-5P in EPB41L3-3′UTR were illustrated in Figure 9E. As shown in Figure 9F, there was no significant difference in the relative luciferase activity between EPB41L3-3′UTR-Mut+ miR-452-5P and EPB41L3-3′UTR-MUT+ NC groups (*P*>0.05), but the relative luciferase activity of EPB41L3-3′UTR-WT+ miR-452-5P group was significantly decreased when compared with EPB41L3-3′UTR-WT+ NC group (*P*<0.01).

### miR-452-5P was involved in the LINC00052-regulated expression of EPB41L3

To clarify whether miR-452-5P was involved in the LINC00052 mediated expression of EPB41L3, the EPB41L3 mRNA and protein expression levels were measured by RT-qPCR and western blot assay. After a several complex transfection, the pcDNA3.1-LINC00052+miR-452-5P inhibitor group had a higher expression of EPB41L3 mRNA than pcDNA3.1-LINC00052+miR-452-5P(*P*<0.01), while the si-LINC00052+miR-452-5P group had a lower expression of EPB41L3 mRNA than si-LINC00052+miR-452-5P-inhibitor group (*P*<0.01)(Supplementary Figure 2A). The similar results of EPB41L3 protein expression were found in western blot analysis (Supplementary Figure 2B). All of the above results indicated that LINC00052 might downregulate the expression of EPB41L3 by binding miR-452-5P.

## DISCUSSION

LncRNAs could regulate protein-coding genes at transcriptional, post transcriptional and epigenetic levels, and they play pivotal roles in tumor physiological processes. Increasing evidence demonstrated that lncRNAs were closely related to tumorigenesis, metastasis, diagnosis or prognosis, acting as roles of tumor suppressor genes or oncogenes [28–32].

Our previous studies revealed that LINC00052 was downregulated in HCC and LINC00052 could downregulate the expression of NTRK3 by miR-128 and miR-485-3p to strengthen the invasion and migration of HCC cell lines [11]. But Salameh A, *et al*. reported that LINC00052 was upregulated in breast cancer and promoted cancer growth through HER3 signaling [33]. LINC00052 has been shown to serve as either tumor suppressors or oncogenic factors, with their specific functions depending on diverse biological processes. In this study, we found that LINC00052 was downregulated in HCC tissues and HCC cell lines. In addition, we identified the function of LINC00052 in HCC cells. LINC00052 depletion could promote cell proliferation, colony formation, migration and invasion in HCC cell lines *in vitro* and our previous studies demonstrated that LINC00052 knockdown promoted tumor growth and metastasis *in vivo* [11]. Thus, LINC00052 holds great promise as a novel diagnostic and prognostic marker for HCC.

Through transcriptome microarray analysis, we found that EPB41L3 was downregulated in A554 cells compared with SMMC7721 cells. We further found that overexpression of LINC00052 activated EPB41L3 expression, and knockdown of LINC00052 downregulated EPB41L3 expression *in vitro* (Figure 2). Immunohistochemistry assay showed weak or no expression of EPB41L3 in the metastasizing liver and lung of mice in the LINC00052 knockdown group *in vivo*. Accumulating evidence indicated that EPB41L3 inhibited tumor cell migration, invasion and served as tumor suppressor [34–37]. In this study, we first reported EPB41L3 was down-regulated in HCC tissues and HCC cell lines. EPB41L3 inhibited HCC cell migration and invasion *in vitro* and *in vivo*, which was consistent with other studies. However, the underlying mechanisms of LINC00052 upregulating EPB41L3 to inhibit tumor cell migration and invasion remains unclear.

Recently, accumulated evidences show that there is a novel regulatory mechanism between lncRNAs and miRNAs. LncRNA may function as competing endogenous RNA (ceRNA) sponges binding to miRNAs, reciprocal repress the expression and biological functions of miRNAs and their target genes [26, 38–40]. For example, HULC acted as an endogenous sponge to downregulate the miR-372 expression in liver cancer [26]. UCA1 could act as an endogenous sponge by directly binding to miR-216b and downregulating miR-216b expression in hepatocellular carcinoma cell [38]. Metastasis-associated lung adenocarcinoma transcript 1 (Malat1) modulates serum response factor (SRF) through miR-133 as a competing endogenous RNA and established a novel connection among Malat1, miR-133, and Srf in myoblast differentiation [41]. TUG1 enhanced tumor-induced angiogenesis and VEGF expression via directly binding to the miR-299 [42].

To elucidate the molecular mechanism of LINC00052 inhibiting tumor cell migration and invasion, bioinformatics analysis was performed to explore the potential targeted gene of LINC00052. Firstly, we found the expression of miR-452-5P was increased in A554 cells (Figure 6A), overexpression of miR-452-5P reduced LINC00052 expression, whereas inhibition of miR-452-5P expression resulted in a significant upregulation of LINC00052, suggesting that LINC00052 and miR-452-5P could form a reciprocal repression feedback loop. Furthermore, we provided evidence that miR-452-5P targeted LINC00052 by directly binding to two miRNA-binding sites in LINC00052 sequence and miR-452-5P mimic could abolish the luciferase activity of LINC00052 by luciferase assays, suggesting that LINC00052 was negatively regulated by miR-452-5P. These data indicated that LINC00052 might be a target of miR-452-5P.

MiR-452 was shown to be overexpressed in urothelial carcinomas [25]. MiR-452 was enriched in neural crest cells, was sufficient to rescue Dlx2 expression in Dicer mutant pharyngeal arches, and regulated non cell autonomous signaling involving Wnt5a, Shh and Fgf8 that converged on Dlx2 regulation in PA1, knockdown of miR-452 *in vivo* decreased *Dlx2* expression in the mandibular component of PA1, leading to craniofacial defects [43]. MiR-452-5P was up-regulated and predicted poor patient survival and advanced TNM stage in HCC patients, and miR-452 was identified to promote cancer stem cells by inhibiting Sox7 through activating Wnt/β-Catenin signaling pathway [21]. Zheng *et al*. showed that miR-452 expression was significantly increased in HCC tissues and HCC cell lines, overexpression of miR-452dramatically accelerated proliferation, and significantly promoted HepG2 and QGY-7703 cells migration and invasion *in vitro* and miR-452 directly targets the 3′-untranslated region of cyclin-dependent kinase inhibitor 1B (CDKN1B), ectopic miR-452 expression suppressed CDKN1B expression on mRNA and protein level [44]. In this study, we found the expression of miR-452-5P was increased in HCC tissues and HCC cell lines, overexpression of miR-452-5P remarkably promoted proliferation, migration and invasion *in vitro*, knockdown of miR-452-5P obtained the opposite results, which was consistent with other studies. Furthermore, our results confirmed that EPB41L3 was one of the target genes of miR-452-5P, overexpression of miR-452-5P reduced the expression of EPB41L3, whereas inhibition of miR-452-5P increased the expression of EPB41L3, luciferase assays confirmed that EPB41L3 was a direct target of miR-452-5P.

Although miR-452 has been experimentally shown to target a large number of protein-coding genes, our study found that miR-452-5P also targeted LINC00052. In this study, we investigated whether miR-452-5P mediated the suppressive effects of LINC00052 in oncogenesis. Our results indicated that although overexpression of LINC00052 suppressed tumor cell proliferation, migration and invasion, upregulated miR-452-5P could largely rescue these effects (Figure 8). Furthermore, we found that LINC00052 knockdown combined with miR-452-5P overexpression most significantly reduced the expression level of EPB41L3(Supplementary Figure 2). These data indicated that LINC00052 downregulated the expression of EPB41L3 to inhibit tumor development by binding miR-452-5P.

In summary, our data demonstrated that LINC00052 acted as a tumor suppressor by inhibiting malignant progression of human HCC and revealed a novel LINC00052-miR-452-5P-EPB41L3 regulatory network in HCC. LINC00052 may be considered as a potential target for the HCC therapies based on downregulating EPB41L3 and deserves further investigation in the future.

## MATERIALS AND METHODS

### Cell culture and human tissue samples

Human hepatocellular carcinoma (HCC) cell lines SMMC7721, SK-hep1, Huh7, HepG2; human immortalized, normal human liver cell line (L02); and the embryonic kidney cell line 293T were obtained from the Chinese Academy of Sciences Cell Bank. They were cultured in Dulbecco's Modified Eagle Medium (DMEM) of high glucose with 10% fetal bovine serum (FBS, BI, ISR). All cells were incubated at 37°C in a humidified incubator with 5% CO_2_. Twelve pairs of primary HCC and adjacent non-tumor tissues were obtained from patients undergoing surgery at the affiliated hospital of Guizhou medical university. Fresh tissue samples were collected and processed within 10 min. Each sample was snap-frozen in liquid nitrogen and then stored at −80°C. The data do not contain any information that could identify the patients. All patients provided written informed consent and ethical consent was granted from the Committees for Ethical Review of Research involving the affiliated hospital of Guizhou medical university (Guizhou, China).

### Transient transfection

Transfections were performed using the Lipofectamine 3000 kit (Invitrogen, Carlsbad, CA, USA) according to the manufacturer's instructions. Double-stranded microRNA oligonucleotides mimic or inhibitors or small interfering RNAs and their respective negative control RNAs were transfected into cells at 75 pmol per well of six-well plate according to the manufacturer's instructions, while 2.5 μg plasmids per well were transfected for DNA. The microRNA mimic, inhibitors and small interfering RNAs were purchased from GenePharma (Shanghai, China). The cells were harvested 48h after transfection. The sequences of the RNA used in transfections were indicated in Supplementary Table 2.

### Reverse transcription and quantitative real-time PCR (RT-qPCR)

Total RNA were extracted from cells using Trizol reagent (Life Technologies Corporation, Carlsbad, CA, USA). RNA concentration and quality were determined by the 260/280 nm ratio using a NanodropSpectrophotometer (ND-2000, Thermo, USA). FastStart essential DNA Green Master (Roche, Indianapolis, IN, USA) was used for RT-qPCR. U6 and GAPDH were used as internal controls. All results were expressed as the means ± sd of at least three independent experiments. Comparative quantification was determined using the 2^−ΔΔCt^ method. The primers used are presented in Supplementary Table 2.

### Cell proliferation assay

Cell proliferation assays were performed using CellTiter 96® Aqueous One Solution Cell Proliferation Assay (MTS, Promega, USA). After transfection, cells were seeded in 96-well plates at the density of 2000/well. 20 μL of MTS were added into per well at different time points(0h, 24h, 48h, 72 h) and incubated at 37°C for 2 h. The absorbance was measured at 490 nm. The cell proliferation curves were plotted using the absorbance at each time point.

### Colony-formation assay

Twenty-four hours post-transfection, cells were seeded into six-well plates (1×10^3^cells/well) and cultured for 14 days. Colonies were visualized by a crystal violet cell colony staining kit and the number of colonies was counted.

### Wound-healing assay

Cells were seeded in 6-well plates at a density of 5 × 10^5^ cells. Upon reaching confluence, the cells were serum starved in FBS-free medium for 12h. A wound was produced by scraping across the cell monolayer using a 200μl sterile polystyrene micropipette tip. The cells were cultured and allowed to migrate into the denuded area for 48h. The wounds adjacent to the lines were photographed prior to and 24 and 48h after scratching under a phase contrast microscope using a 20× objective lens. Cells were counted using ImagePro Plus 6.0 software in the marked area between the labeled lines and the wound.

### Cell migration and invasion assay

24-well chambers with 8 μm pore size (Costar 3422; Corning, NY, USA) were used in cell migration and invasion assays. Cells (5×10^4^) in 100 μl of serum-free media were seeded into the upper chamber (without or pre-coated with 500 ng/ml Matrigel solution (BD, Franklin Lakes, NJ, USA) in migration or invasion assay separately), 600 μL of 10% FBS medium was placed in the lower chamber. After 48h of incubation, the upper chambers were removed from the plates and cells on the top side of the chamber were wiped with a cotton swab. Migrating or invading cells were fixed and then stained by Giemsa staining. Five randomly fields were counted under a microscope and photos were taken.

### Western blot analysis

Total proteins were extracted from the cells using RIPA buffer with protease inhibitors (Beyotime, Shanghai, China) on ice, subjected to SDS-PAGE and electrophoretically transferred to PVDF membranes (Merck Millipore, MA, USA). Membranes were incubated in 5% non fat milk dissolved in Tris-buffered saline (TBS) containing 0.1% Tween-20 for 2h at room temperature and then incubated with primary antibodies as follows: EPB41L3 (1:500, Abcam, Cambridge, MA, USA), GAPDH (1:2000, Proteintech, Wuhan, China), followed by incubation with appropriate correlated HRP-conjugated secondary antibody. Then the membranes were incubated with secondary antibodies (Proteintech, Wuhan, China) at room temperature for 2h. Immunoblots were visualized by enhanced chemiluminescence (ECL kit, Advansta, USA) and scanned using ECLTM chemiluminescence detection system (Pierce, USA).

### Immunohistochemistry assays

All tissues were fixed with 10% paraformaldehyde, and embedded in paraffin wax. Paraffin sections were placed in incubators kept at 55°C for 4h. The sections were immersed in two consecutive washings in xylol for 20 min to remove paraffin. Sections were then hydrated with different concentrations of ethanol including 100%, 95%, 85%, 70% and deionized water, respectively. The sections were immersed in citrate buffer solution (0.01 mol/L, pH 6.0) and heated to repair antigen, then 0.5%Triton-x-100 was incubated 30 min after washing with PBS. Slides were imaged under a light microscope (Olympus, Japan) at ×400 magnification.

### Construction of stable cell lines

To obtain cell lines stably expressing EPB41L3, SMMC7721 cells were transfected with the plasmid pcDNA3.1-EPB41L3(Genscript, Nanjing, China) and plasmid pcDNA3.1 respectively, selected with neomycin (1000 μg/ml) for 4 weeks. The short-hairpin RNA targeting human EPB41L3 were ligated into the pGreenPuro shRNA vector (SBI, USA) according to the manufacturer's protocol. Transfected SMMC7721 cells were selected with puromycin (1μg/ml) for 4 weeks. Selected cells were further subcloned for uniform stable cell lines. The overexpression and the silence efficiency were analyzed using RT-qPCR and western blot.

### Reporter vectors construction and luciferase assays

LINC00052 full length and EPB41L3-3′UTR sequences were amplified by PCR and cloned into a PGL3-control (Promega, Madison, WI, USA) to construct luciferase reporter vector (LINC00052-WT and EPB41L3-WT). The sequence of putative binding site (miR-452-5P) was replaced as indicated LINC00052-MUT and EPB41L3-MUT to mutate the putative binding site of LINC00052 or EPB41L3. SMMC7721 or HEK-293T cells were seeded in 96-well plates and the cells were co-transfected with LINC00052-WT (or LINC00052-MUT) or EPB41L3-WT (or EPB41L3-MUT) and miR-452-5P mimic or miR scrambled NC when they reached 50-70% confluence. The luciferase activities were measured at 48h after transfection by Dual-Luciferase reporter assay kit (Promega, Madison, WI).

### Animal studies

The male BALB/c nude mice (4-6 weeks old) were purchased from Laboratory Animal Services Center of CQMU. 20 mice were randomly allocated into four groups. A total of 1×10^7^ SMMC7721 cells stably overexpressing EPB41L3 (pcDNA3.1- EPB41L3), or cells down-expressing EPB41L3 (sh-EPB41L3) and their control cells were injected into the liver tissue of mice, respectively. After 6 weeks, mice were killed for intrahepatic metastasis assessment.

### Statistical analysis

Statistical analyses were performed using SPSS 17.0 software (SPSS, Chicago, IL, USA). Differences between two groups were assessed using Student's *t*-test (two-tailed). Each experiment was performed at least three times. Results of experiments are displayed as mean ± standard deviation. A *P* value<0.05 was considered to indicate statistical significance.

## SUPPLEMENTARY MATERIALS TABLES AND FIGURES


