# Supplementary material for: LINC00052 upregulates EPB41L3 to inhibit migration and invasion of hepatocellular carcinoma by binding miR-452-5p
Source: Oncotarget. 2017 Jun 29;8(38):63724–37. doi: 10.18632/oncotarget.18892 (PMC5609956; doi:10.18632/oncotarget.18892)
Supplement: Supplementary file 1 [file oncotarget-08-63724-s001.pdf]

LINC00052 upregulates EPB41L3 to inhibit migration and invasion of hepatocellular carcinoma by binding miR-452-5p

SUPPLEMENTARY MATERIALS

Supplemental Table 1: A part of downexpressed genes from microarray data (A554_vs_SMMC7721)

Probe name	Fold change	Regulation	Genbank accession	Genomic coordinates	Gene symbol
A_32_P206479	19.3076115	down	NM_178457	chr20:57833989-57834048	ZNF831
A_23_P336796	2.6859296	down	NM_173601	chr12:42476664-42476605	GXYLT1
A_33_P3313250	5.4132619	down	NM_001004477	chr1:158548776-158548717	OR10X1
A_23_P257231	2.339464	down	NM_031279	chr4:109663325-109663266	ETNPPL
A_23_P50697	8.060045	down	NM_006905	chr19:43372365-43372306	PSG1
A_33_P3363271	9.9658268	down	NM_000724	chr10:18829909-18829968	CACNB2
A_24_P288424	3.3262763	down	NM_016045	chr20:57608816-57608757	SLMO2
A_24_P879740	2.7200704	down	NM_005909	chr5:71504756-71504815	MAP1B
A_23_P35912	2.073339	down	NM_033306	chr11:104815551-104815492	CASP4
A_23_P212050	6.1779046	down	NM_000055	chr3:165490878-165490819	BCHE
A_33_P3290709	2.9466036	down	NM_001167890	chrX:13651616-13651675	EGFL6
A_23_P4536	28.4491176	down	NM_012307	chr18:5393451-5393392	EPB41L3
A_23_P251293	3.6400983	down	NM_003087	chr10:88719878-88722412	SNCG
A_23_P23611	2.0642496	down	NM_001008219	chr1:104207021-104207080	AMY1C
A_23_P59718	2.0801708	down	NM_003130	chr7:87835338-87835279	SRI
A_24_P99090	2.1373438	down	NM_018204	chr13:53035863-53035922	CKAP2
A_23_P36905	2.6991798	down	NM_001507	chr13:49796299-49796358	MLNR
A_24_P98555	2.1462071	down	NM_207009	chr10:120877058-120877117	FAM45A
A_24_P945283	12.4661312	down	NM_021120	chrX:69725254-69725313	DLG3

Supplemental Table 2: Real-time PCR, construction plasmid primer, and sequences of shRNA, siRNAs, miRNA mimics and inhibitors

**Real-time PCR primer**	
LINC00052	F: AGCTCTCTCACCATGCGATT
	R:TGTTTGCAGACTGTAGGGCT
GAPDH	F: GGTCTCCTCTGACTTCAACA
	R:GTGAGGGTCTCTCTCTTCCT
U6	F:GCTTCGGCAGCACATATACTAAAAT
	R:CGCTTCACGAATTTGCGTGTCAT
EPB41L3	F:TTGCAGCATTACCTCCCGAT
	R: ACTGTCACTCGGGTCATTGT
miR-452-5P	GGCCCCACGCAAAGAAGAAGG
miR -548-5P	GCAAAAGTAATTGCGGTTTTTGCC
miR-4672	GCTTACACAGCTGGACAGAGGCA
miR-4712-5P	CGCTCCAGTACAGGTCTCTCATTTC
miR-4774	GCGCCGTCTGGTATGTAGTAGGTAATAA
miR-217	GCTACTGCATCAGGAACTGATTGGA
miR-187	TCGTGTCTTGTGTTGCAGCCC
miR-218	ATGGTTCCGTCAAGCACCATG
**Construction plasmid primer**	
PGL3-LINC00052	F: GCTCTAGAACTGGTAGTTGTTGACATTTCTG
	R: GCTCTAGACAAGAACTTGAAGATGTGCCATT
PGL3-EPB41L3 3'UTR	F: GCTCTAGATAGAAGCTGTCTAGGTCCGTC
	R: GCTCTAGAAGAGCTTTCAGTGCATTTGCC
**Sequences of shRNA, siRNAs, miRNA mimics and inhibitors**	
sh-EPB41L3	F: GATCCGCCGGGAGAGTTTGAACAATTCTTCCTGTCAG
	AAATTGTTCAAACTCTCCCGGC TTTTTG
	R:AATTCAAAAAGCCGGGAGAGTTTGAACAATTTCTG
	ACAGGAAGAATTGTTCAAACTCTCCCGGCG
sh-NC	F:GATCCGATGAAATGGGTAAGTACATTCAAGAGATG
	TACTTACCCATTTCATCTTTTTG
	R:AATTCAAAAAGATGAAATGGGTAAGTACATCTCTT
	GAATGTACTTACCCATTTCATCG
si-LINC00052	UUAUUCACAUCACUGCAUGTT
hsa-miR-452-mimic	F: AACTGTTTGCAGAGGAAACTGA
	R:AGTTTCCTCTGCAAACAGTTTT
Negative control(NC)	F: GUACCUGACUAGUCGCAGATT
	R:UCUGCGACUAGUCAGGUACTT
Has-miR-452-inhibitor	UUGACAAACGUCUCCUUUGACU
Negative control inhibitor(NC-in)	CAGUACUUUUGUGUAGUACAA

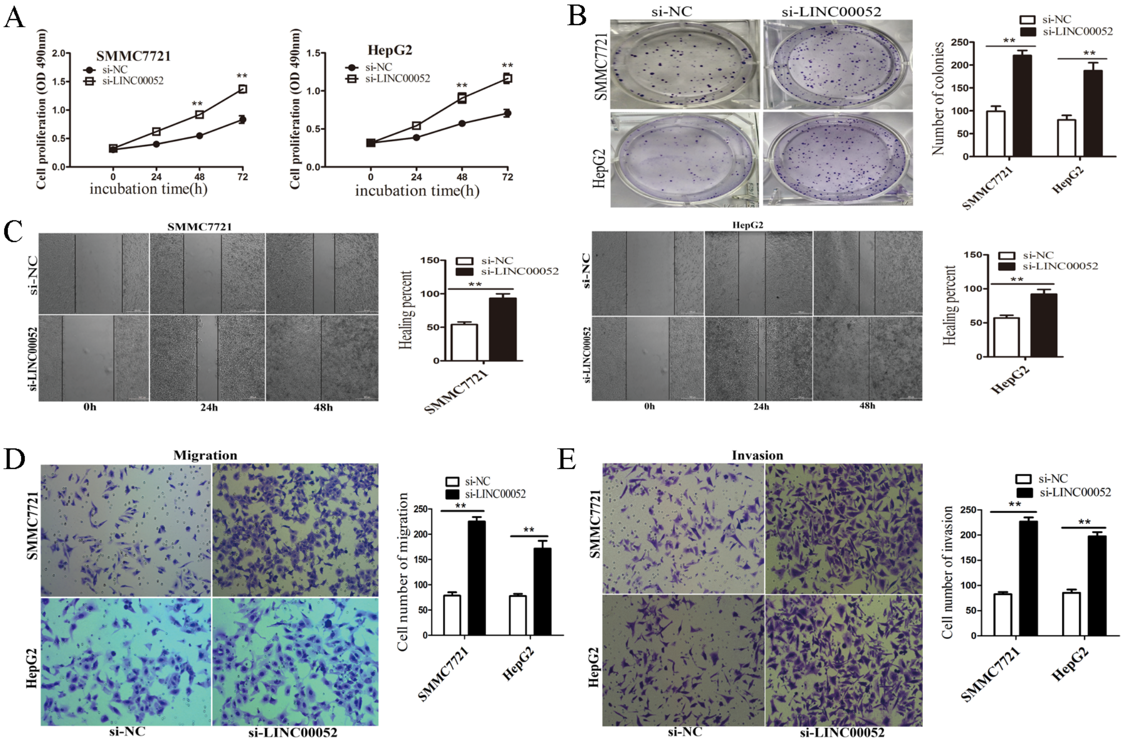

Supplementary Figure 1: Knockdown of LINC00052 promoted HCC cell proliferation, migration and invasion. (A) Growth curves of SMMC7721 and HepG2 cells after transfection with si-LINC00052 or si-NC. ***P*<0.01. **(B)** Colony formation assays for determining the effect of LINC00052 upregulation on the growth of SMMC7721 and HepG2 cells. Representative graphs are shown. The data graphs depict the count number from three independent experiments. ***P*<0.01. **(C)** Wound healing assay for determining the effect of LINC00052 upregulation on the healing of SMMC7721 and HepG2 cells. ***P*<0.01. **(D, E)** Transwell and invasion assay of LINC00052 overexpressed cells. Data are shown as mean ± s.d. (n = 3) and are representative of three independent experiments. Scale bars = 100 μm. ***P*<0.01.

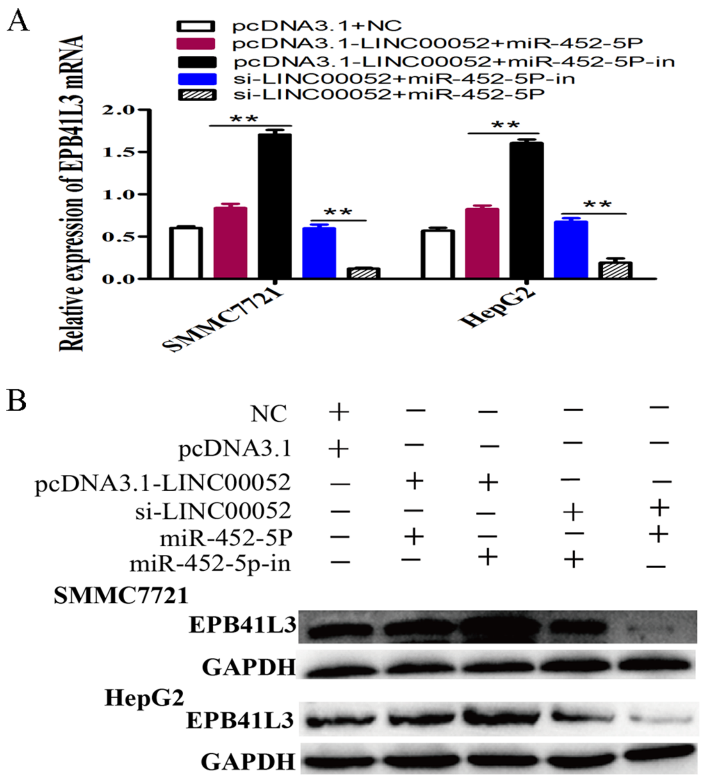

Supplementary Figure 2: miR-452-5P was involved in the LINC00052 regulated expression of EPB41L3. (A) RT-qPCR analysis of for LINC00052 and miR-452-5P regulated EPB41L3. Transcript levels were normalized to GAPDH expression. **(B)** Western blot analysis of LINC00052 and miR-452-5P regulated the expression of EPB41L3, using GAPDH as endogenous control. **P<0.01.
